# Meta-analysis of a megafish: assessing patterns and predictors of Alligator Gar movement across multiple populations

**DOI:** 10.1186/s40462-025-00544-7

**Published:** 2025-03-10

**Authors:** Hayden C. Roberts, Florian J. Kappen, Matthew R. Acre, Daniel J. Daugherty, Nathan G. Smith, Joshuah S. Perkin

**Affiliations:** 1 Department of Ecology and Conservation Biology, Texas A&M University, 2258 TAMU, College Station, TX 77843 USA; 2 Texas Parks and Wildlife Department, Heart of the Hills Fisheries Science Center, 5103 Junction Highway, Mountain Home, TX 78058 USA; 3 Present Address: U.S. Geological Survey, Columbia Environmental Research Center, 4200 E. New Haven Road, Columbia, MO 65201 USA

**Keywords:** Fish movement theory, Movement ecology, Restricted movement paradigm, Environmental associations, Scale, Riverine fish, Leptokurtosis

## Abstract

**Background:**

Freshwater megafishes are among some of the most commercially and ecologically important aquatic organisms yet are disproportionately threatened with range and population reduction. Anthropogenic alterations of rivers influencing migrations are among the most significant causes for these declines. However, migratory fishes do not always respond similarly to movement barriers and thus it is necessary to develop models to predict movements of freshwater migratory fishes in the face of anthropogenic alteration. Predicting movement of freshwater fishes is often investigated using statistical packages. However, empirical studies assessing these packages have led to mixed results, questioning its applicability to all taxa. We argue that spatial, temporal, and environmental attributes are more influential for movement of a migratory megafish, the Alligator Gar (*Atractosteus spatula*), than the current parameters explored in a globally relevant fish movement model.

**Methods:**

This study explored two independent mobile telemetry datasets investigating Alligator Gar movement on the Brazos and Trinity rivers. Environmental associations were investigated to predict Alligator Gar displacement and dispersal using generalized additive models, generalized linear models, and model selection. Leptokurtosis of Alligator Gar populations was also assessed. Predictability of the movement model was tested by comparing observed to model derived stationary and mobile components making up a leptokurtic movement distribution.

**Results:**

Our study suggests that current and antecedent measures of discharge and water temperature are positively correlated with Alligator Gar displacement and dispersal. However, these patterns are only detectable when monthly relocation intervals are explored rather than seasonal scales. Leptokurtosis was observed in both Alligator Gar populations. However, movement was normally distributed (i.e., mesokurtic) under tracking events following high flood pulses. Additionally, predicted Alligator Gar movement was significantly farther under modeled values compared to observed values, in part because the species undergoes cyclical migrations for reproduction that are sensitive to water temperature and discharge.

**Discussion:**

In conclusion, this study provides an alternative framework to assess the movement patterns of migratory fishes, which could be tested on additional freshwater fishes, and suggests that assessing spatial, environmental, and temporal processes simultaneously are necessary to capture the complexities of fish movement which currently are unavailable for the movement model we investigated.

**Supplementary Information:**

The online version contains supplementary material available at 10.1186/s40462-025-00544-7.

## Background

A persistent issue for many freshwater aquatic organisms is the decline of populations due to land modification and habitat fragmentation [1]. Of particular concern are large-bodied aquatic organisms such as freshwater megafauna, defined as organisms able to reach at least 30 kg in mass [2]. Megafauna species range and population abundance are disproportionately reduced compared to smaller bodied species where declines in freshwater megafauna fishes (hereafter “megafishes”) are most severe [2]. The coupling of certain intrinsic life history characteristics with anthropogenic disturbances are considered major drivers for these declines. Megafishes often exhibit a periodic life history strategy, meaning they are long lived, mature later in life, and exhibit migratory behavior [3–5]. Given that megafishes are ecologically and economically important, as they are often keystone or indicator species and support commercial and recreational fisheries, it is critical to further investigate the effects of anthropogenic alteration on their persistence for the conservation of freshwater ecosystems and sustainable procurement of fisheries resources [6].

Dam construction often results in habitat fragmentation, impeding fish migration. Furthermore, dams can reduce floodplain connectivity and modify downstream river hydrology [7, 8]. Considering that discharge is positively associated with upstream migratory movement in fishes [9], it is likely that hydrologic alteration of dams influence the rate, timing, and magnitude of migratory fish movement in downstream habitats. Despite this, fishes react to dam construction in several ways. Many neotropical fishes alter their movements in the presence of dams by conducting shorter reproductive migrations to occupy spawning habitat upstream or downstream of reservoirs [10, 11]. Alternatively, Blue Sucker (*Cycleptus elongatus*) populations of central Texas were documented suspending spawning migrations due to altered hydrologic regimes from upstream dams [12]. The effect of dams on fish migrations can also lead to different outcomes within the same species. One example is with Chinook Salmon (*Oncorhynchus tshawytscha*), where mitigation practices (e.g., fish passage structures) along dams of the Snake River led to higher recruitment and spawner abundance in salmon populations compared to those in the upper Columbia River, where dams do not have fish passage structures [13]. Therefore, assessing the movement dynamics of additional species exhibiting migratory behavior warrants further investigation, especially when populations may have different hydrologic scenarios due to dam construction.

In lotic systems, movement of freshwater fishes is often conceptualized by the restricted movement paradigm (RMP). Originally proposed by [14] and extended by [15] and [16], the RMP posits that movement by stream fishes that do not engage in obligatory migrations (e.g., diadromous or long-distance spawning migrations) is heterogenous and composed of stationary and mobile components. Within a population, the majority of fish move very little, while a few travel long distances. This heterogeneous movement behavior in stream fishes is often described by a leptokurtic distribution. Under this model, the stationary component represents a high frequency of short, centralized movements, creating a sharp peak. The mobile component, by contrast, captures rare, long-distance movements, resulting in longer tails than those in a Gaussian (i.e., mesokurtic) distribution. From this [17], developed a fish movement package in R to model components of the RMP (e.g., leptokurtosis) using relatively few parameters. The Radinger and Wolter model (RW model) suggests parameters such as fish total length (TL), caudal fin aspect ratio (AR), stream order (SO), and study duration can predict fish dispersal, along with stationary and mobile components of fish populations [18, 19]. Many studies have corroborated assumptions and tested the utility of the RW model with mixed results. One assumption of the RW model is that diffusive spread exists among stream fishes, which has been detected by many field studies [20–22]. Additionally, observed stationary and mobile components of fish populations have been compared to those reported by the RW model. Both [23] and [24] reported an order of magnitude underestimation between modeled stationary and mobile components compared to observed values. The former study [23] interpret their findings as a lack of understanding of small-bodied Leuciscid movement patterns, particularly in large rivers. However, we argue that a more cohesive explanation may be because the RW model does not account for basic environmental and spatial components that can influence fish movement, nor how time between movement observations may further influence these relationships. Given the temporal stochasticity of environmental conditions (e.g., discharge) in many temperate streams, the relationship between fish movement and these conditions may be dampened as time between relocations increases because larger time intervals exclude more temporal variation that may affect movement. Therefore, an assessment of the RW model and movement dynamics of fishes incorporating these processes may be useful in advancing stream fish movement theory, especially for large migratory megafishes in need of conservation and management initiatives.

Currently, movement by the Alligator Gar (*Atractosteus spatula*) remains untested by the RW model, as do other megafishes. With maximum weights exceeding 100 kg and lengths over 2 m, this megafish, like many others, has extinction prone life history characteristics including specific habitat requirements for reproduction, increased longevity, and variable year class strength [25–27]. Once widely distributed across much of the Mississippi and other Gulf of Mexico drainages, reproducing populations of Alligator Gar are primarily limited to states such as Texas, Louisiana, and Arkansas, along with portions of eastern Mexico [28]. Range reductions stem from targeted eradication [29], reproductive habitat loss due to modification of floodplains, and river fragmentation from dam construction [28]. Globally considered vulnerable [30], the species also has special interest in Texas as it is a Species of Greatest Conservation Need due to increasing interest in recreational angling and understanding of the species’ role as a top predator in ecosystems [31–32].

Several studies suggest Alligator Gar exhibit migratory behavior. According to [33], Alligator Gar inhabiting the Brazos River migrated over 20 river kilometers (rkm) into tributary streams when high flow pulses connecting floodplains coincided with optimal temperature conditions for reproduction (20–30°C). Additionally [25], observed that movements of tagged Alligator Gar were highest during the spawning season, defined as May and June in [34], with some movements exceeding 80 rkm. Furthermore, a separate study on the Brazos River [35] detected leptokurtosis while investigating Alligator Gar populations of the lower Brazos River and thus theoretically could be modeled accurately by the RW model. Considering these studies, and the sensitivity of Alligator Gar movement to environmental variables, an assessment of Alligator Gar movement using the RW model and how it may be related to environmental, spatial, and temporal components is therefore warranted.

**Fig. 1:**
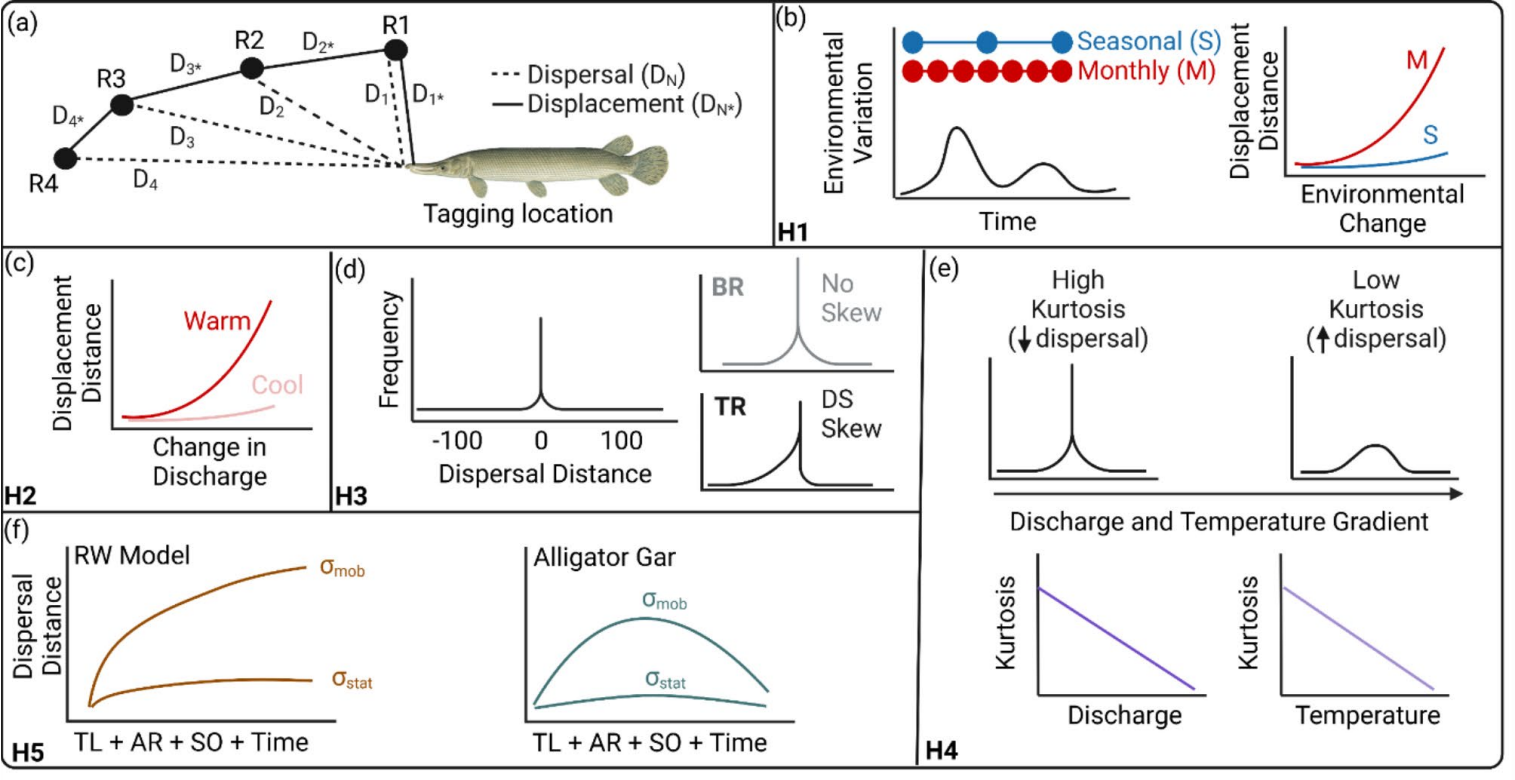
Overview of concepts tested in our meta-analysis. Bold text in the bottom left corner of panels b-f indicate the hypothesis illustrated. In (**a**), movement can be measured as *dispersal* (D_N_) in which the distance from each successive relocation (R1 – R4) is compared to the original tagging location, or *displacement* (D_N*_) in which the distance from a successive relocation is compared to the previous relocation (e.g., R2 versus R3). In (**b**), the temporal resolution of relocations might be seasonal (S) or monthly (M) and the level of resolution has implications for detecting relationships between environmental variation and displacement. In (**c**), alignment between water temperature and changes in discharge can stimulate greater fish movement during warm periods. In (**d**), dispersal of freshwater fishes is typically expressed as a leptokurtic distribution of movements from the tagging location in upstream (positive numbers) or downstream (negative numbers) directions where skewness will be inconsequential in the Brazos River (BR) and demonstrate downstream bias in the Trinity River (TR) due to the arrangement of impoundments. In (**e**), high kurtosis values are indicative of limited dispersal while lower kurtosis values represent greater dispersal, and kurtosis might decline (i.e., greater movement) as water discharge or temperature increases. In (**f**), the RW model developed by [17] predicts dispersal distance by estimating stationary (σ_stat_) and mobile components (σ_mob_) of fish populations based on fish total length (TL), caudal fin aspect ratio (AR), stream order (SO), and the length of time between tagging and relocation (time). The migratory behavior of Alligator Gar (*Atractosteus spatula*) posits deviations in components from expected values. Figure created with BioRender.com (https://biorender.com/) using Alligator Gar image generated by Rick Hill (with permission)

The goal of our study was to investigate movement dynamics of Alligator Gar in two independent river basins and test the predictability of the RW model on dispersal patterns. Using two different datasets of fish tagged in the Trinity and Brazos rivers of Texas, our objectives were to: (1) assess how environmental variables influence a change in Alligator Gar location (i.e., displacement) across studies and different relocation intervals: (2) test for leptokurtosis and assess differences in dispersal magnitude and skewness across datasets: (3) determine whether kurtosis (i.e., the distribution of dispersal data) is related to environmental variables such as temperature and discharge: and (4) assess the presence of diffusive spread and applicability of the RW model in predicting Alligator Gar movement across datasets by comparing model outputs to observed data. Our meta-analysis tested several hypotheses assessing Alligator Gar displacement and dispersal (Fig. 1a). We hypothesize that longer time intervals between successive relocations will reduce statistically distinguishable effects of environmental variables on fish displacement (H1; Fig. 1b) where the magnitude of displacement predicted by a change in discharge will depend on water temperature (H2; Fig. 1c). Additionally, leptokurtosis will be present in each population, but differences in movement magnitude and skewness will differ due to different hydrological scenarios of the study sites (H3; Fig. 1d). We also predict that kurtosis will decrease in the presence of higher flows and temperatures, as Alligator Gar are more mobile when these values increase (H4; Fig. 1e). Lastly, we expect that diffusive spread will not be present in Alligator Gar populations, as migratory behavior posits a cyclical return to locations after spawning, leading to an overestimation of movement parameters from the RW model (H5; Fig. 1f). An assessment of these patterns across multiple Alligator Gar populations will help determine whether the species responds similarly to environmental conditions across river basins with varying degrees of hydrological alteration and provide crucial information for management agencies to determine whether responses to environmental change for this imperiled megafish are generalized or context dependent.

## Methods

### Study area

**Fig. 2:**
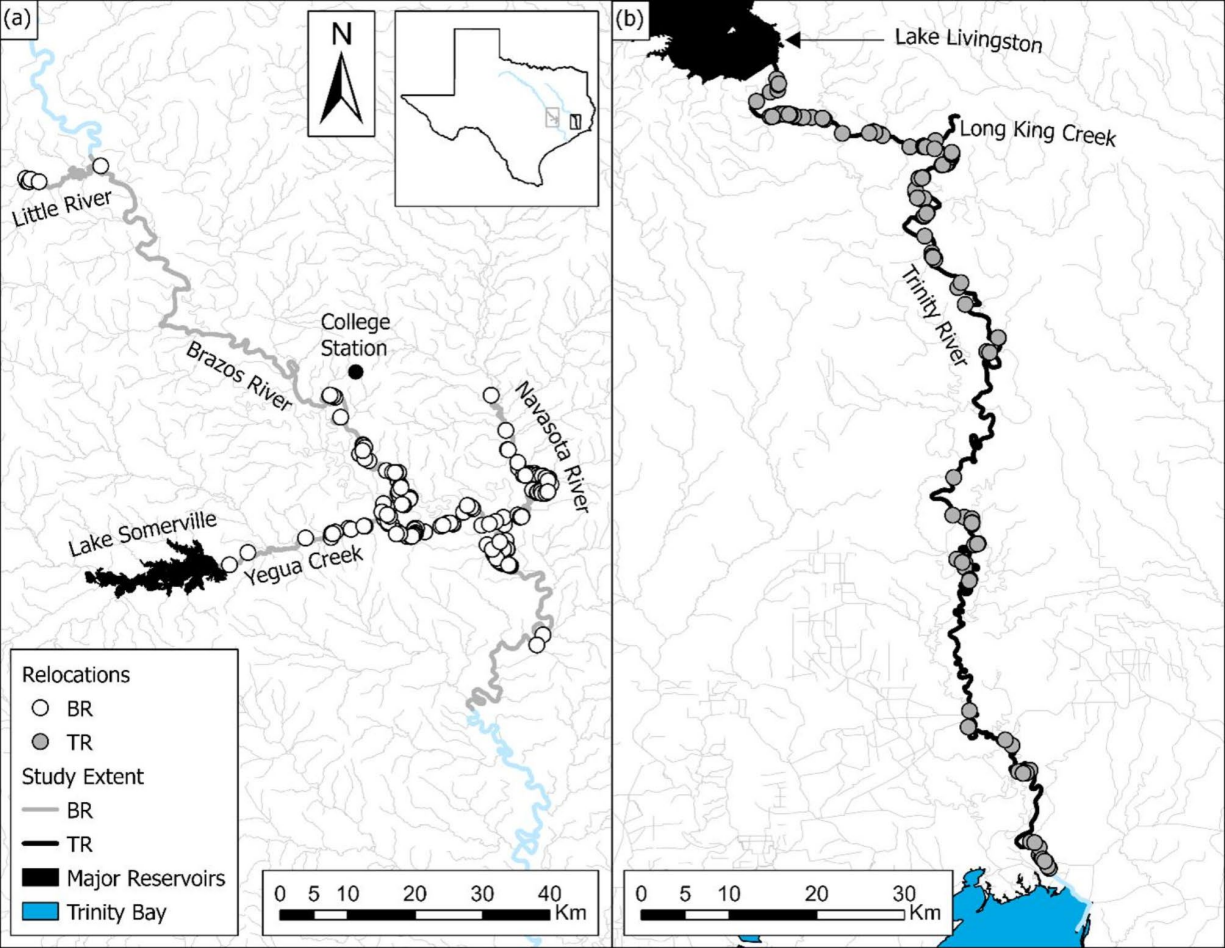
Study areas for Alligator Gar (*Atractosteus spatula*) telemetry research on the Brazos River (BR) and Trinity River (TR), along with major tributaries in each. Manual tracking data were obtained from datasets explored in [33] and [25], respectively (i.e., BR and TR study). In (**a**), the extent of the BR study is in grey with white circles denoting manual telemetry relocations. In (**b**), black lines represent the extent of manual tracking in the TR study with grey circles denoting manual telemetry relocations. Where the light blue line begins in (**b**), a lock and dam structure near Wallisville, Texas acts as a saltwater barrier to prevent salinity intrusion into the lowermost Trinity River at its mouth in Trinity Bay. Black shaded areas denote major reservoirs in each study including Lake Somerville in (**a**) and Lake Livingston in (**b**)

The Brazos River originates near the border of New Mexico and Texas flowing 2,060 rkm southeast into the western Gulf of Mexico [36]. Downstream of Waco, Texas (TX), the lower Brazos River is a low gradient, meandering river flowing through a predominantly agricultural watershed with several major tributaries including the Navasota River, Yegua Creek, and Little River [37]. Major reservoirs upstream of the study area include Lake Somerville on Yegua Creek, Lake Whitney on the Brazos River, and Lake Limestone on the Navasota River. The Brazos River study [33] assessed Alligator Gar movement across 362 rkm of water (200 rkm mainstem, 162 rkm tributaries) centralized around College Station, TX (Fig. 2a). The Trinity River is the longest river completely within Texas, flowing 1,140 rkm from its headwaters just south of the Oklahoma border to Trinity Bay of the western Gulf of Mexico. Downstream of Lake Livingston Dam, the lower Trinity River is a meandering lowland river surrounded by the pine forests of eastern Texas. The Trinity River study [25] assessed Alligator Gar movement across 177 rkm starting immediately downstream of Lake Livingston on the Trinity River down to 3 rkm from the river mouth where a lock and dam structure acts as a saltwater barrier (Fig. 2b).

### Tagging and tracking

Our meta-analysis used mobile tracking data from the Brazos and Trinity River studies (i.e., BR and TR studies). Tagging and tracking methods are summarized below and described in detail in [33] and [25]. Alligator Gar were captured using experimental gill nets (76–127 mm bar mesh) that targeted deep pool habitats with little to no flow [38]. The TR study also used rod-and-reel and juglines for collecting Alligator Gar. Both studies used acoustic transmitters (Models CT-82-2-E and CT-82-2-I; Sonotronics, Tucson, Arizona) while the BR study also implemented combined acoustic and radio-transmitter (CART) tags (model MM-RC-16-25; Lotek, Ontario, Canada). Transmitters were surgically inserted into the peritoneal cavity in the BR study and attached to the base of the dorsal fin in the TR study.

Mobile tracking methods from each study were also similar. Tagged gar were relocated by boat traveling at approximately 8 km/h downstream towing a Sonotronics omnidirectional hydrophone. A directional Sonotronics hydrophone was also used to separate multiple signals of tags and pinpoint individual fish locations. For the BR study, standardized tracking regions [33] were surveyed at predominantly monthly intervals. Additional tracking was conducted when flows increased, allowing access to ephemeral tributaries, floodplains, and oxbow lakes. This resulted in a total of 15 tracking events from May of 2020 to August of 2021 (Table 1). For the TR study, mobile tracking of the entire study area occurred at seasonal intervals (approximately 3 months) with additional tracking occurring opportunistically at 1–2-month intervals when flooding allowed access to ephemeral habitats similar to the BR study. This resulted in nine tracking events from March 2009 to July of 2010 (Table 1). Global positioning system (GPS) coordinates for tagging locations and relocations were logged in the field to estimate Alligator Gar displacement and dispersal distances. Distances between Alligator Gar locations were determined by snapping GPS coordinates to river polylines implemented in ArcGIS Pro (version 3.0.0, Esri). Coordinates were then read into R and the *riverdist* package [39] was used to determine the hydrographic distances for both displacement and dispersal observations.

**Table 1:** Summary of tracking events from [33] and [25]

Event	Date Start	Date End	Scale	Relocations	Study
1	5/11/2020	5/14/2020	M	11	BR
2	6/2/2020	6/12/2020	M	20	BR
3	6/17/2020	6/17/2020	M	7	BR
4	9/10/2020	9/26/2020	S	18	BR
5	10/7/2020	10/16/2020	M	16	BR
6	11/4/2020	11/12/2020	M	16	BR
7	12/4/2020	12/4/2020	M	3	BR
8	1/13/2021	1/20/2021	M	19	BR
9	2/22/2021	3/3/2021	M	16	BR
10	3/24/2021	4/2/2021	M	30	BR
11	4/21/2021	5/3/2021	M	27	BR
12	5/26/2021	6/3/2021	M	14	BR
13	6/23/2021	7/2/2021	M	23	BR
14	7/22/2021	7/24/2021	M	22	BR
15	8/23/2021	8/26/2021	M	17	BR
1	3/10/2009	3/11/2009	M	21	TR
2	4/6/2009	4/8/2009	M	29	TR
3	4/27/2009	5/19/2009	M	16	TR
4	6/3/2009	6/3/2009	M	9	TR
5	8/13/2009	8/15/2009	S	33	TR
6	11/2/2009	11/4/2009	S	7	TR
7	1/25/2010	1/27/2010	S	28	TR
8	4/27/2010	4/28/2010	S	8	TR
9	7/6/2010	7/8/2010	S	14	TR

The top table provides information on the Brazos River (BR) study, while the bottom table summarizes the Trinity River (TR) study. In each, the start and end dates of each tracking event, tracking group, and number of Alligator Gar relocations are reported. The scale column refers to whether individuals relocated between tracking events were at monthly (1–60 days) or seasonal (61–120 days) intervals, abbreviated M and S, respectively

### Environmental data

For our first two hypotheses (H1-H2) we were interested in how proximal and antecedent conditions of discharge (cubic meters per second) and water temperature (°C) were related to Alligator Gar displacement. The daily average discharge (*Q*) from the nearest U.S. Geological Survey (USGS) gage [40] was determined for each day a fish was tagged or relocated (see Table S1 for all gages used in this study). We calculated change in daily average discharge (*ΔQ*) as the difference in values between the day of the focal relocation and day of the previous relocation. If a fish moved in closer proximity to a different USGS gage due to a long movement on the mainstem or movement into a tributary from the mainstem, a new USGS gage was used to represent discharge for the following focal and successive relocations (see Figure S1). For each observation, the directional change in discharge (i.e., rise or fall in magnitude) was transformed to an absolute value and z-score (*z*) transformed such that unit mean and variance was standardized across gages of different stream orders. For water temperature, values were acquired from temperature loggers (Onset HOBO^®^, Onset Computer Corp., Bourne, Massachusetts) deployed as a part of the original studies and measured at 1–3 h intervals (1 h for BR study, 2–3 h for TR study). The average daily water temperature (*T*) and change in average daily water temperature (*ΔT*) for each day a fish was tagged or relocated was then determined using the same methods described for discharge. However, *T* was not *z* transformed, as a two-tailed t-test reported that water temperature was not significantly different across studies (*t* = -1.17, *p* = 0.25).

For dispersal (H3-H4), data were investigated across tracking events. Therefore, the number of observations was directly tied to the number of tracking events across each study, as we were interested in determining how antecedent conditions of discharge and temperature relate to kurtosis values. To assess environmental correlates with dispersal (H4), 30-day average discharge values from the last relocation in a tracking event were calculated and similarly *z* transformed. However, only a single reference USGS gage [40] in this analysis for each study was used (USGS 08108700 for BR; USGS 08066500 for TR). Average monthly temperature values were determined from the same temperature loggers and calculated similarly to monthly discharge values. Considering daily temperature values across the study sites were not significantly different, monthly temperature was also not *z* transformed.

### Statistical analysis

#### Hypothesis 1

To test our first hypothesis (H1), all displacement data were split into two categories based on the temporal resolution between consecutive relocations, including the approximation of monthly (M; 1–60 days between relocations) and seasonal (S; 61–120 days between relocations) intervals. Intervals > 120 days were excluded from the analysis due to low sample size in the BR study (*N* = 1). Data were filtered for outlier observations to mitigate development of unrealistic standard error estimates in our models (Figure S2). We considered displacement observations outliers if they exceeded a *z* of five (i.e., five standard deviations from mean displacement). Our filtering strategy retained all data from the M and S intervals except two displacement observations of Fish 138 exceeding 130 rkm (*z* > 8.5) from the Brazos River. We fit a series of non-monotonic generalized additive models (GAMs) with a negative binomial error distribution for exploring independent effects of *ΔQ*, *Q*, *T*, and *ΔT* on displacement. For each model, a smoothing function was fit for the independent variable and allowed to vary by interval (M or S), while also including the additive effect of *study*. GAM models were fit using the ‘gam’ function from the *mgcv* package [41]. We concluded support for H1 if a greater number of significant relationships occurred for the M interval compared to the S interval. Preliminary results of this analysis revealed the M interval produced the strongest relationships (see *H1: Temporal scale* results section). Therefore, all subsequent analyses excluding H5 focused only on the M interval.

#### Hypothesis 2

Using data from the M interval, we tested for temperature-dependent relationships between displacement and *ΔQ* using a series of GAMs and monotonic generalized linear models (GLMs). These models were parameterized using displacement distance as the response variable, *ΔQ* and *T* on the day of relocation as continuous predictor variables, and the identity of the *study* river as a categorical fixed effect. We modeled GLM and GAM relationships using a negative binomial error distribution and developed seven competing models for each. These seven models included: (1) an intercept-only model, (2) the individual effect of *T*, (3) the individual effect of *ΔQ*, (4) a two-way interaction between *ΔQ* and *T*, (5) additive effects model between *ΔQ* and *T*, (6) a three-way interaction between *ΔQ*, *T*, and *study*, and (7) a two-way interaction between *ΔQ* and *T* and the additive effect of *study*. We ranked models using the Akaike information criterion for small sample size (AICc) according to [42] and used the ‘model.sel’ function from the *MuMIn* package in R [43]. The results of the top-ranked model were illustrated using partial effects plots, which provided support for H2 if the top model reported Alligator Gar displacement being greatest when temperatures were highest and discharge changed the most.

#### Hypothesis 3

Leptokurtic dispersal distributions and upstream versus downstream skewness were tested using pooled and tracking-event-specific data. To ensure the same fish were modeled in each tracking event, data were filtered to follow cohorts of tagged fish, while only considering observations from the M interval. We modeled the movement of 23 Alligator Gar tagged in May and June of 2020 from the BR study and 27 Alligator Gar tagged in February of 2009 from the TR study. Leptokurtosis for pooled data and tracking events across studies was tested using the Anscombe Glynn test implemented with the ‘anscombe.test’ function from the *moments* package in R [44]. This test assesses significant deviation from a mesokurtic distribution with a kurtosis (*K*) value of 3, with distributions characterized by *K* < 3 termed platykurtic and distributions characterized by *K* > 3 termed leptokurtic. We assessed the skewness of pooled datasets using a D’Agostino test implemented using the ‘agostino.test’ also using the *moments* package and concluded support for H3 existed if leptokurtic distributions were present and movement skewness varied by study.

#### Hypothesis 4

Using the same dataset from H3, relationships between dispersal and 30-day averages for *Q* and *T* were tested using GAMs. We built independent models for *Q* and *T*, along with an interaction between the two and combined data from both studies to assess tracking-session-specific kurtosis values as a function of 30-day averages of *Q* (*Q*_*z−30d*_) and *T* (*T*_°C−30d_). GAMs were fit with the ‘gam’ function from the *mgcv* package in R using a negative binomial error distribution, kurtosis as the response variable, and smoothing functions for *ΔQ*_*z−30d*_, *T*_°C−30d_, or their interaction, as the independent variables. We concluded support for H4 occurred if kurtosis declined as discharge and temperature increased and if a significant interaction was present.

#### Hypothesis 5

We tested whether the RW model by [17] predicted dispersal of Alligator Gar by comparing expected and observed dispersal distances for stationary and mobile components of Alligator Gar populations. This involved using all mobile tracking data from both BR and TR studies, as smaller sample sizes led to unreasonable estimates of observed stationary and mobile components. Expected dispersal estimates were derived using the ‘fishmove’ function from the *fishmove* package in R [17]. This function uses fish total length (TL), caudal fin aspect ratio (AR), stream order (SO), and time as predictor variables to estimate the expected distance moved for members of the stationary and mobile components assuming 67% of fish are stationary and 33% are mobile [17]. This model was parameterized using the average TL and median days from tagging across all tracking intervals. The caudal fin AR was kept constant at 1.77 for all tracking events based on measurements collected from images of live fish collected from the Navasota River. A SO of seven was used for the BR study (ComID = 5559352) and six for the TR study (ComID = 1515411), derived from the National Hydrography Dataset [45]. We used the ‘fishmove.estimate’ function from the *fishmove* package to summarize observed dispersal data obtained from each tracking event and study. This function creates a fitted mean estimate and 95% confidence interval for each tracking event from a vector of dispersal distances obtained from field observations. We elected to perform 500 pseudoreplicates to estimate dispersal distances for stationary and mobile components based on the superimposed double-normal distributions as described in [17]. We concluded there was support for H5 if observed dispersal distances and their confidence intervals fell below the 95% confidence intervals of expected dispersal distances. All analyses were conducted in R, version 4.2.1 [46] using α < 0.05 for statistical significance.

## Results

### Telemetry and movement data summary

From the BR study, 43 Alligator Gar (1180–2380 mm TL) were tagged and relocated at least once from May 2020 to August 2021, resulting in 259 manual relocations. From the TR study, 46 Alligator Gar (800–2130 mm TL) were tagged and relocated at least once from March 2009 to July 2010, leading to 185 manual relocations. Eighteen of the TR relocations included fish encountered more than once in a tracking event and were removed from analysis, reducing our sample size to 167 manual relocations. For the BR study, mean dispersal across all data (*n* = 259) was 11.3 rkm while mean displacement was 6.1 rkm. However, one individual (i.e., Fish 138) tagged at the mouth of Yegua Creek made several movements between this tributary and the Little River, leading to several dispersal observations over 140 rkm. Of the 167 manual relocations from the TR study, average dispersal was 10.0 rkm while average displacement was 9.4 rkm. Several long-distance movements were also observed in the TR study including a 89 rkm dispersal observation and a 77 rkm displacement observation.

### H1: temporal scale

**Fig. 3:**
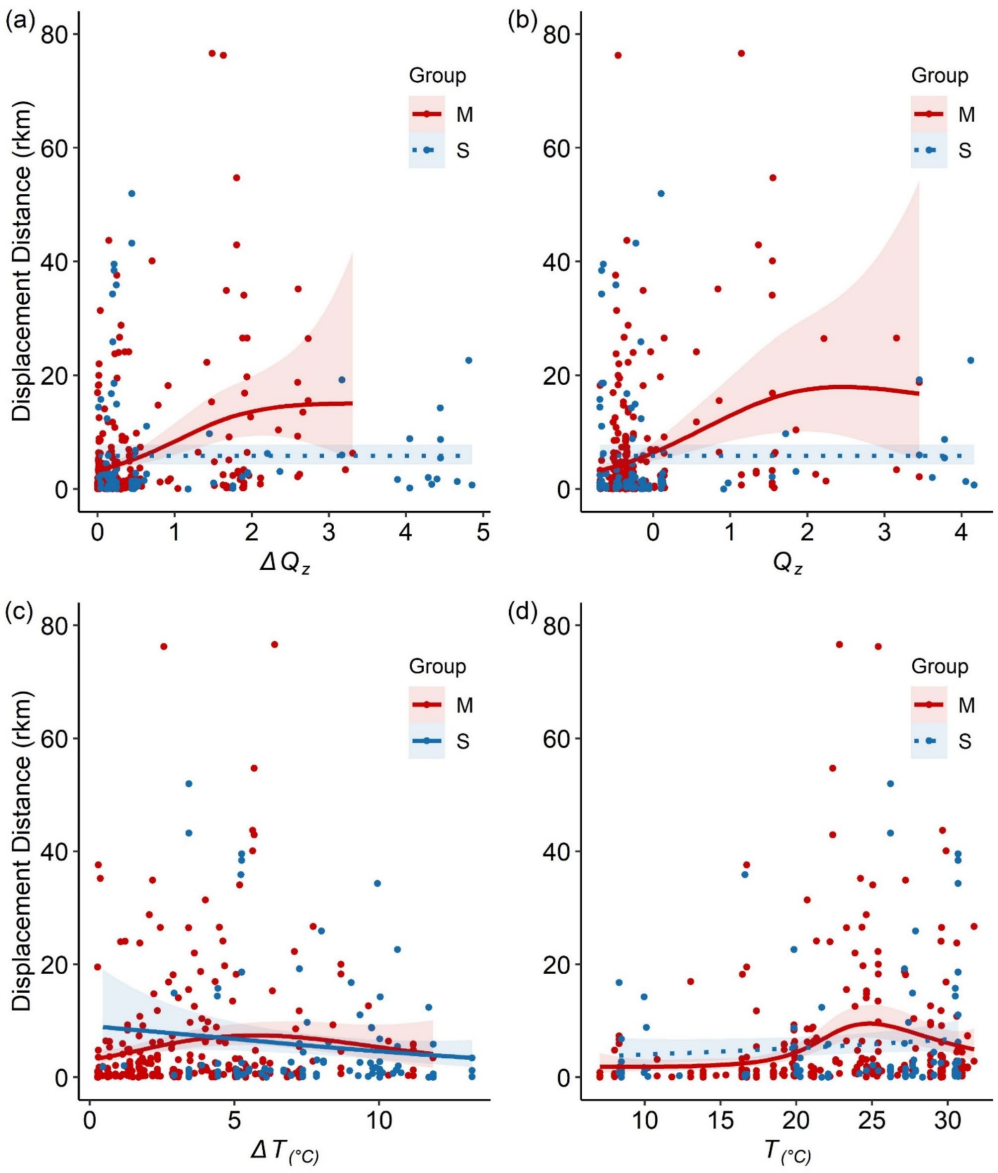
Generalized additive models illustrating the effect of time interval on environmental variables influencing Alligator Gar (*Atractosteus spatula*) displacement distance (rkm). Solid lines represent a significant relationship (*p* < 0.05) while dotted lines denote non-significance (*p* > 0.05). Red lines model data at the monthly (M) time interval (1–60 days between relocations) while blue lines model the seasonal (S) time interval (61–120 day between relocations). In (**a**), the relationship between the change in z-score (*z*) transformed daily average discharge (Δ*Q*_*z*_) and displacement distance are illustrated. In (**b**), the relationship between *Q*_*z*_ and displacement distance are illustrated. In (**c**), the relationship between the change in daily average water temperature (*ΔT*_°C_) and displacement distance is given. In (**d**), the relationship between *T*_°C_ and displacement distance is given. Shaded regions for each line denote 95% confidence intervals

Tests for the effects of temporal scale on relationships with environmental variables predicting displacement revealed stronger patterns at the M interval than the S interval. Across all models, there was a significant effect of *study* (Table 2). The M interval smoothing functions yielded significant relationships for each parameter, while the S interval was only significant when considering the *ΔT* parameter (Table 2). The model with the strongest fit included the relationship between displacement and *ΔQ* (Fig. 3a; Adjusted *R*^*2*^ = 0.11), followed by *Q* and displacement (Fig. 3b; Adjusted *R*^*2*^ = 0.10), which demonstrate that as *ΔQ* or *Q* increase, so does displacement distance, but only for the M interval. The relationship between displacement and *ΔT* resulted in our weakest model (Fig. 3c; Adjusted *R*^*2*^ = 0.02). The relationship between displacement and *T* explained less variation than the discharge models (Fig. 3d; Adjusted *R*^*2*^ = 0.08). For both models, the M interval demonstrated positive correlations with displacement, but not for the S interval, similar to the discharge models.

**Table 2:** Parameter and smoothing function estimates from generalized additive models assessing the Temporal effect of relocation interval on environmental associations with Alligator Gar (*Atractosteus spatula*) displacement

Parameter Estimates						Smoothing Functions		
Model	Parameter	Estimate	SE	*Z*	*p*	Parameter	*χ* ^*2*^	*p*
*ΔQ*	Intercept	8.44	0.09	90.27	**< 0.01**	ScaleM	37.49	**< 0.01**
	StudyTR	0.36	0.16	2.30	**0.02**	ScaleS	0.01	0.92
*Q*	Intercept	8.42	0.09	93.14	**< 0.01**	ScaleM	29.69	**< 0.01**
	StudyTR	0.41	0.16	2.54	**0.01**	ScaleS	0.26	0.78
*ΔT*	Intercept	8.54	0.10	87.38	**< 0.01**	ScaleM	13.06	**< 0.01**
	StudyTR	0.40	0.17	2.39	**< 0.02**	ScaleS	6.14	**0.04**
*T*	Intercept	8.36	0.09	94.40	**< 0.01**	ScaleM	48.25	**< 0.01**
	StudyTR	0.48	0.17	2.88	**< 0.01**	ScaleS	3.21	0.07

Each model independently investigated the effect of the change in average daily discharge (*ΔQ*), average daily discharge (*Q*), change in average daily water temperature (*ΔT*), and average daily water temperature (*T*) on displacement and included an additive effect of *study*. For parameter estimates, the coefficient estimate, standard error (SE), test statistic (*Z*), and *p*-values (*p*) are provided. For the smoothing functions, smoothing parameters include data at the monthly (ScaleM) or seasonal (ScaleS) intervals, the Chi-Square test statistic (*χ*^*2*^), and associated *p*-values (*p*). Bold text denotes significant relationships for both parameter estimates and smoothing functions

### H2: temperature-dependent responses to ΔQ

**Fig. 4:**
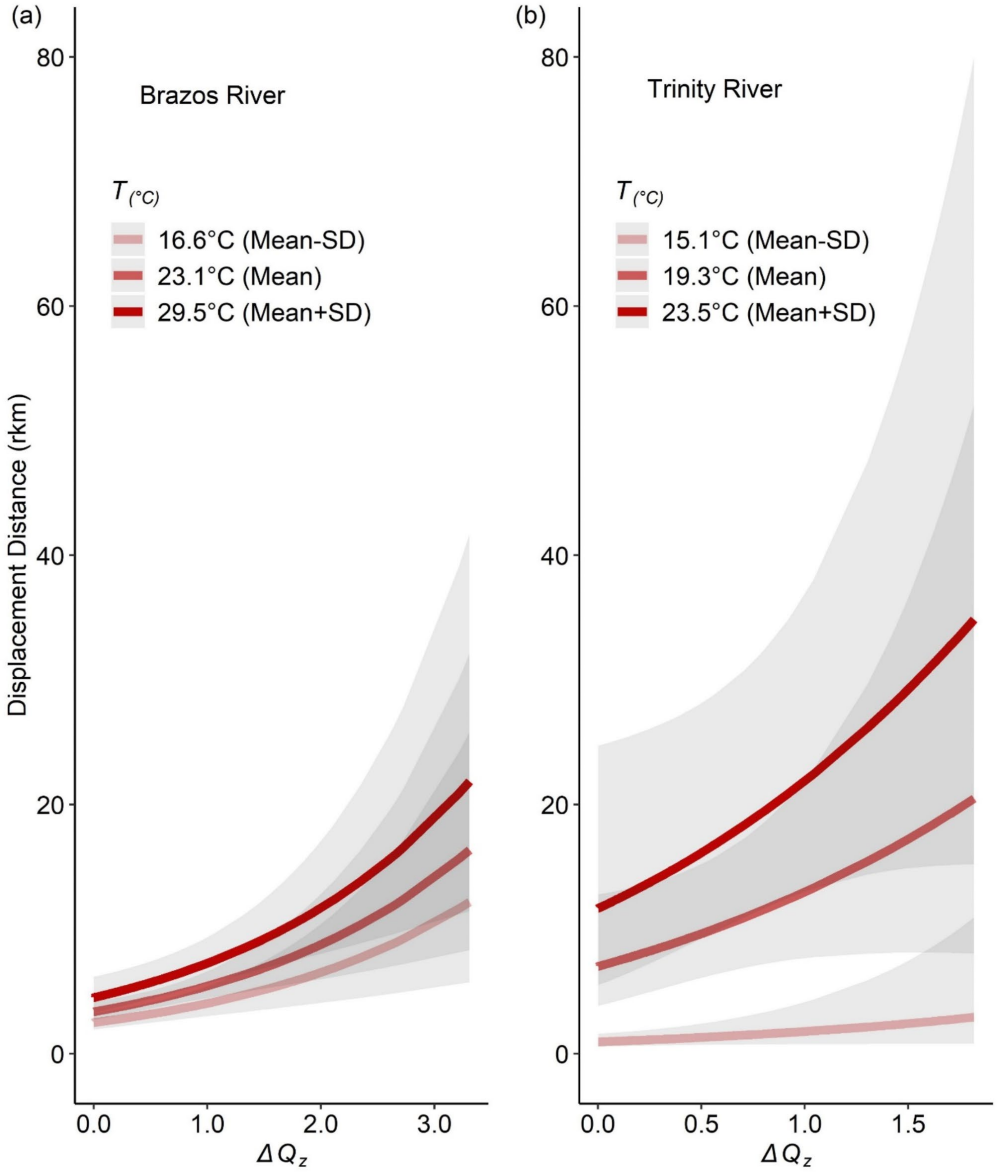
Partial effects from top model assessing predictors of Alligator Gar (*Atractosteus spatula*) displacement distance (rkm) of data subsetted to the M interval (1–60 days between relocations). In (**a**), the change in *z* transformed daily average discharge (*ΔQ*_*z*_) moderated at one standard deviation (SD) below the mean, mean, and one SD above the mean water temperature (*T*_°C_) conditions for the Brazos River are displayed. In (**b**), the same response is given, but for the Trinity River dataset. Warmer colors represent higher water temperatures. Grey shaded regions for each line display 95% confidence intervals

Alligator Gar displacement was positively correlated with *ΔQ* and *T* in both rivers studied, although the pattern varied slightly by river. The top model was a GAM containing a three-way interactive smoothing term between *ΔQ*, *T*, and *study* identity (Table 3). There were no competing models, and this model had an Adjusted *R*^*2*^ = 0.18. Partial effects plots illustrated an increase in displacement distance as *ΔQ* increased, with the highest predicted displacement values observed when *T* was moderated at one standard deviation (SD) above mean conditions in the Brazos (Fig. 4a) and Trinity rivers (Fig. 4b). When *T* was held at one SD below mean conditions there was little change in displacement distance with increases in *ΔQ*, particularly in the Trinity River.

**Table 3:** Comparison of models used to test water temperature-dependent relationships between Alligator Gar (*Atractosteus spatula*) displacement and changes in discharge in the Brazos and trinity rivers

Parameters	Model	K	logLik	AICc	ΔAICc	ω	Adj. *R*^2^
***ΔQ*** * ***T*** * ***Study***	**GAM**	7	-2573.368	**5161.2**	**0**	**0.995**	**0.18**
ΔQ*T + *Study*	GLM	6	-2580.652	5173.6	12.5	< 0.01	0.15
ΔQ*T*Study	GLM	9	-2577.538	5173.7	12.6	< 0.01	0.16
ΔQ*T + *Study*	GAM	5	-2582.834	5175.9	14.7	< 0.01	0.14
ΔQ*T	GLM	5	-2584.214	5178.6	17.5	< 0.01	0.14
*ΔQ* + *T*	GAM	5	-2585.383	5182.4	21.2	< 0.01	0.13
ΔQ*T	GAM	4	-2586.976	5183.8	22.6	0	0.12
*ΔQ* + *T*	GLM	4	-2587.972	5184.1	22.9	0	0.12
*ΔQ*	GAM	3	-2589.96	5188.0	26.8	0	0.11
*ΔQ*	GLM	3	-2592.477	5191.0	29.9	0	0.10
*T*	GAM	3	-2600.295	5208.6	47.5	0	0.06
*T*	GLM	3	-2602.300	5210.7	49.5	0	0.05
Null	GLM	2	-2612.399	5228.8	67.7	0	0.00
Null	GAM	2	-2612.399	5228.8	67.7	0	0.00

Model parameters represent continuous measures of change in daily average discharge (*ΔQ*; z-score transformed) and daily average water temperature (*T*; °C) as well as a categorical factor representing the Brazos River or Trinity River mobile telemetry dataset (*Study*). Candidate model structures included generalized additive models (GAMs) for non-monotonic relationships or generalized linear models (GLMs) for monotonic relationships across seven possibilities: (1) null (intercept only), (2) fixed effect of *T*, (3) fixed effect of *ΔQ*, (4) two-way additive (+) modelling, (5) two-way interactive (*) modelling, (6) two-way interactive modelling and an additive effect, and (7) three-way interactive modelling. The table shows the number of parameters in the model (K), log-likelihood (LogLik), Akaike information criterion score adjusted for small sample size (AICc), the difference in AICc from the top model (ΔAICc), the Akaike weight (ω), and Adjusted R^2^ values. Bolded text denotes the top model

### H3: dispersal modeling

Goodness of fit measures of dispersal data differed across datasets. While investigating all data from the BR study, dispersal data exhibited upstream bias (*skew* = 3.36, *Z* = 11.97, *p* < 0.01). However, there were two outliers revealed in this dataset, Fish 126 and Fish 138 (Figure S3a). Once these individuals were removed, only minimal upstream bias was observed (skew = *0.96*, *Z* = 5.41, *p* < 0.01), as illustrated in Figure S3b and S3c. For the TR dataset, an evaluation of all data yielded significant downstream bias (*skew*=-1.71, *Z*=-6.82, *p* < 0.01) where several fish exhibited significant downstream movements (> 50 rkm) from their tagging locations (Figure S4).

**Fig. 5:**
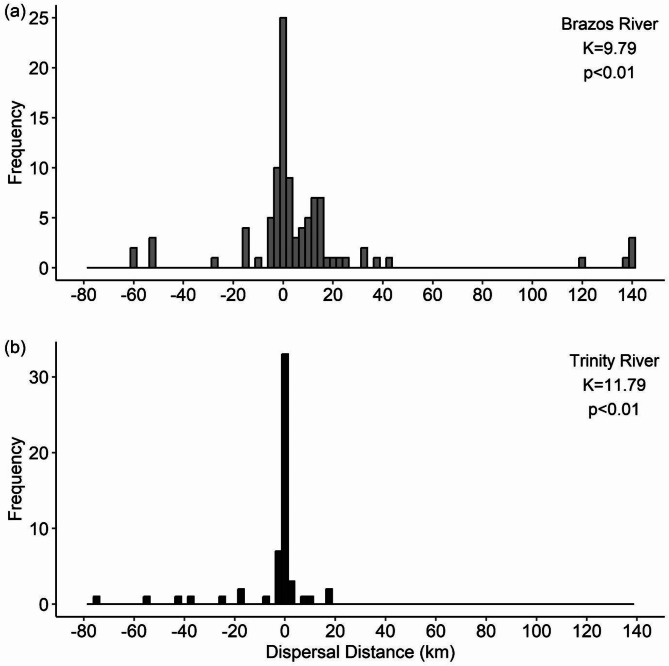
Pooled frequency distributions illustrating the observed kurtosis for Alligator Gar (*Atractosteus spatula*) dispersal distance (rkm) from subsetted individuals of the M interval (i.e., 1–60 day relocation interval). In (**a**), grey bars indicate dispersal frequencies for observations in the Brazos River while in (**b**), black bars indicate values from the Trinity River. For each, the kurtosis value (*K*) and *p*-value are given in the upper right corner. Values where *K* is significantly greater than three suggest a leptokurtic frequency distribution

Kurtosis modeling of subsetted datasets yielded similar results between studies and systems. Under the M interval of select tagged fish investigated from the BR study, the pooled data exhibited a significantly leptokurtic distribution (Table 4; Fig. 5a). The pooled BR data were made up of observations from all tracking events other than events 4, 7, 8, and 13–15, which either had a small sample size (*N* < 6) or did not have observations with a 1–60 day relocation interval. Of the retained BR tracking events, all events were significantly leptokurtic except for events 1, 3, and 12, which displayed a mesokurtic distribution (Table 4). For the TR dataset, our data filtering process yielded four tracking events suitable for analysis (events 1–4). Pooled data of these tracking events were also significantly leptokurtic (Table 4; Fig. 5b). All tracking events were leptokurtic except for event three which was mesokurtic. Histograms illustrating the frequency distributions of each tracking event across studies can be found in Figure S5 and S6.

**Table 4:** Results from Anscombe-Glynn tests assessing kurtosis of Alligator Gar (*Atractostus spatula*) dispersal

Events	K	Z	*p*		Study		*N*	
1	1.56	-1.61	0.11		BR		11	
2	12.80	4.15	**< 0.01**		BR		20	
3	2.71	0.81	0.42		BR		7	
5	9.19	3.59	**< 0.01**		BR		14	
6	9.05	3.57	**< 0.01**		BR		14	
9	5.98	2.95	**< 0.01**		BR		9	
10	5.65	2.80	**< 0.01**		BR		9	
11	5.64	2.69	**< 0.01**		BR		10	
12	1.67	-0.78	0.44		BR		6	
Pooled	9.79	4.85	**< 0.01**		BR		100	
1	14.71	4.38	**< 0.01**		TR		20	
2	7.57	3.15	**< 0.01**		TR		17	
3	2.64	0.40	0.69		TR		11	
4	4.94	2.81	**< 0.01**		TR		7	
Pooled	11.79	4.53	**< 0.01**		TR		55	

In the top half, data are a subset from the cohort of Alligator Gar (*n* = 23) tagged in May and June of 2020 from the BR study [33]. These observations were further filtered such that they only exhibit relocation intervals between 1 and 60 days (i.e., M time interval). The kurtosis (*K*) value for each event retained for analysis is reported, along with the test statistic (*Z*), *p*-value (*p*), and sample size (*N*) for each test. The bottom half denotes Anscombe-Glynn tests on the group of Alligator Gar (*n* = 27) tagged in February of 2009 from the TR study [25] using the same data subsetting strategy. Pooled data represents tests of kurtosis on all the combined tracking events for each study

### H4: kurtosis-environmental relationships

**Fig. 6:**
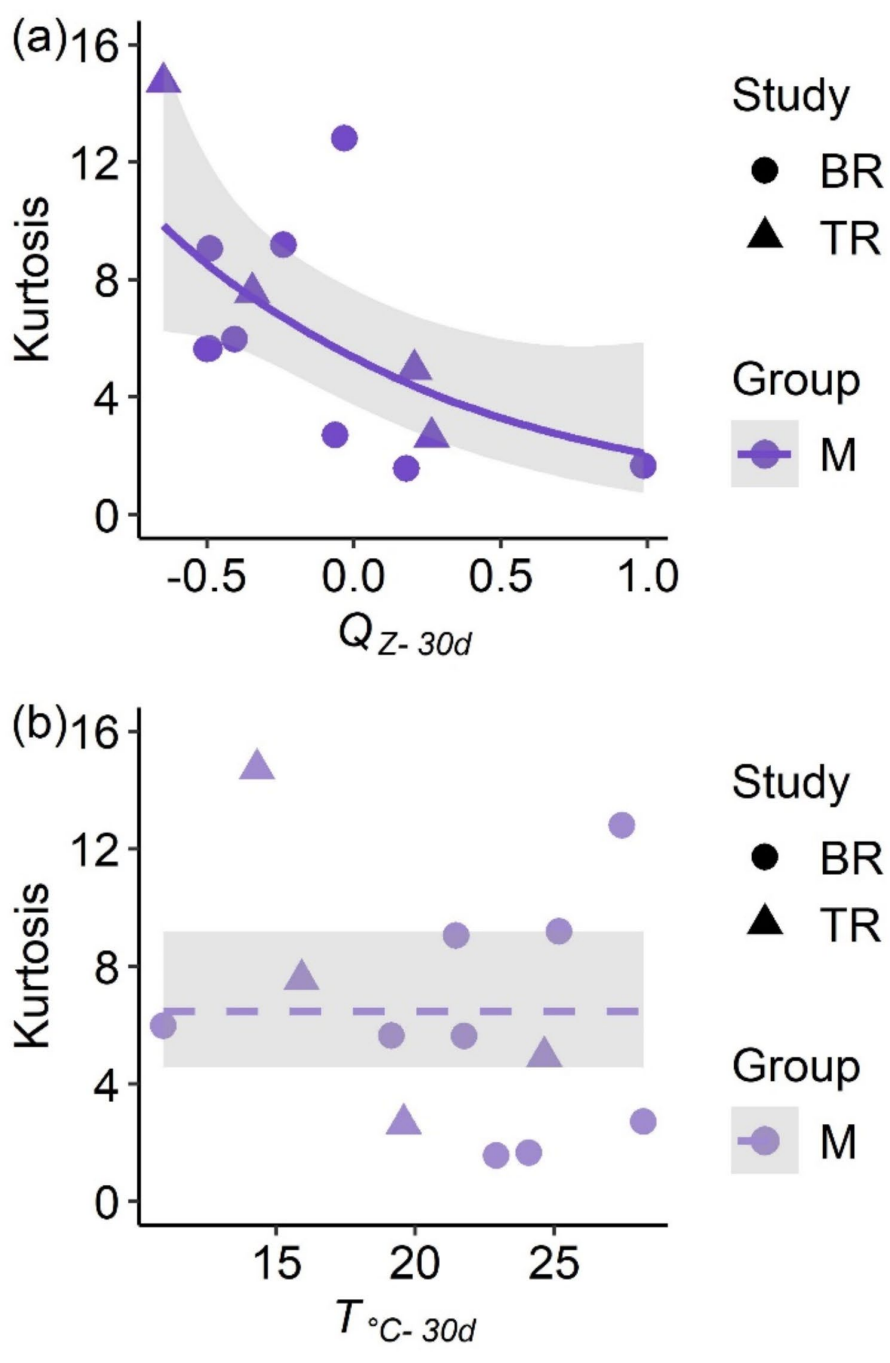
Results from generalized additive models with a negative binomial error distribution assessing environmental variables affecting Alligator Gar (*Atractosteus spatula*) kurtosis values. Data are subsetted such that kurtosis values originate from dispersal data of filtered individuals from the M interval (i.e., 1–60 day relocation intervals). Each observation denotes the kurtosis value for a given tracking event for the Brazos (BR; circles) or Trinity (TR; triangles) rivers. In (**a**), the z-score transformed 30-day average daily discharge (*Q*_*z−30d*_) is plotted against kurtosis values while in (**b**) the 30-day average daily temperature (*T*_°C−30d_) is regressed against kurtosis values. Predictor variables were calculated using the previous 30 days from the last day in each tracking event. Solid lines exhibit a significant relationship (*p* < 0.05) while dashed lines resemble non-significance (*p* > 0.05). Gray shaded regions for each line display 95% confidence intervals

By using the kurtosis values from Table 4 as a response variable, we observed a significant relationship between *Q*_*z−30d*_ conditions and kurtosis values, as revealed by our GAM (*F* = 6.46, *p* = 0.03). This model had moderate fit (Adjusted *R*^*2*^ = 0.31), as illustrated by a significant decrease in kurtosis values as discharge values increased (Fig. 6a). The GAM assessing the individual effect of *T*_°C−30d_ on kurtosis was not significant (*F* = 0.50, *p* = 0.49) and had poor model fit (Adjusted R^2^=-0.04), as illustrated in Fig. 6b. The interaction between monthly discharge and temperature was also not significant (*F* = 3.05, *p* = 0.09), yet it explained a modest amount of variation (Adjusted *R*^*2*^ = 0.25).

### H5: testing RW model

**Fig. 7:**
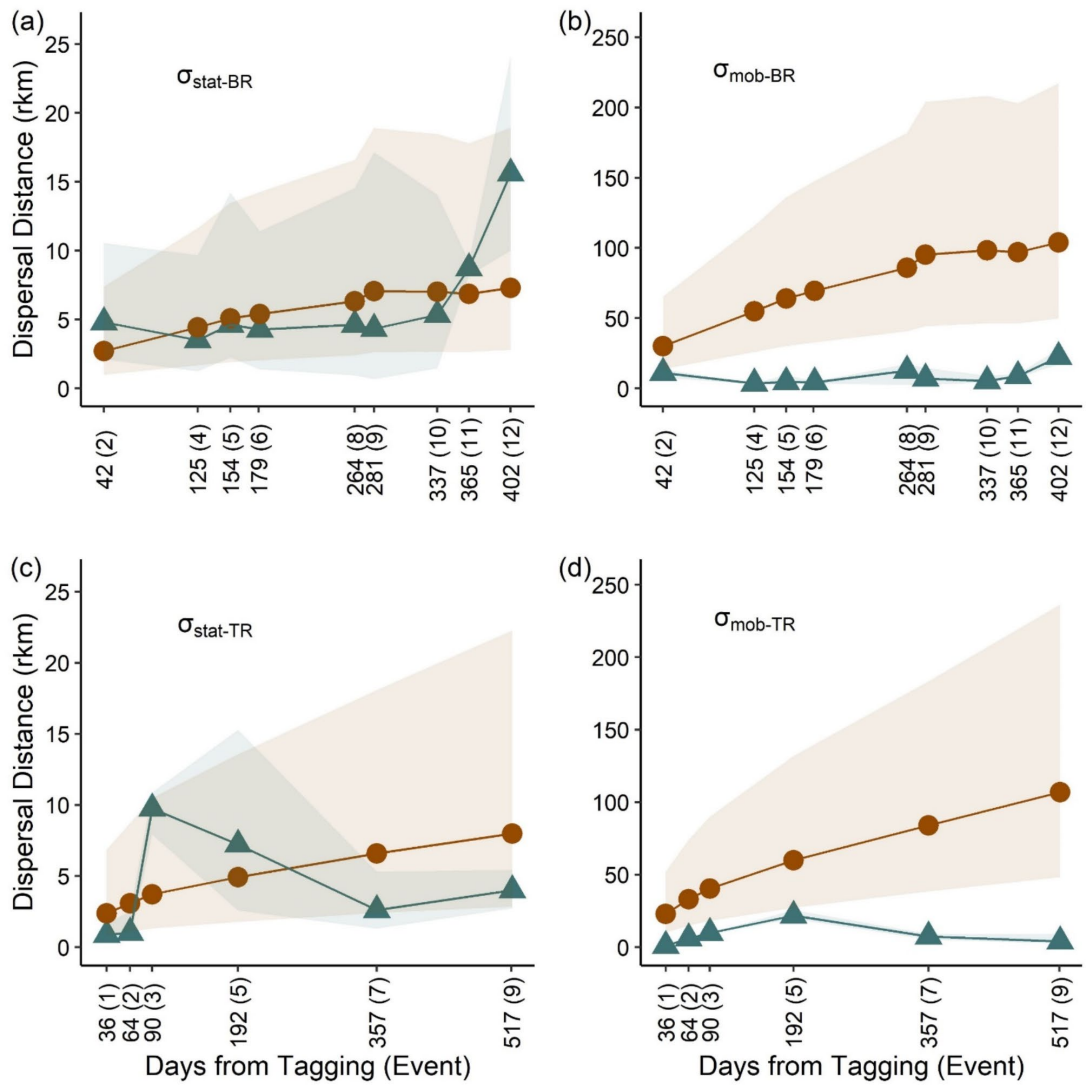
Results from assessing the applicability of the statistical package developed by [17] on determining stationary and mobile components of Alligator Gar (*Atractosteus spatula*) dispersal. In each panel, brown lines with circles exhibit expected model values for each component and study, while dark blue lines with triangles are observed values after performing 500 bootstrap pseudoreplicates from a vector of movement data for each tracking event. Days from tagging represents the median number of days at large for all tagged individuals in each tracking event with the tracking event displayed in parenthesis. Dispersal distance (rkm) represents the stationary or mobile component parameter values selected to predict Alligator Gar movement. In (**a**), the stationary component across time from the Brazos River (σ_stat−BR_) is given while in (**b**), results from the Brazos River mobile component are given (σ_mob−BR_). In (**c**), the stationary component parameter estimates from the Trinity River (σ_stat−TR_) are given while in (**d**), results from the Trinity River mobile component are displayed (σ_mob−TR_)

For both datasets, similar patterns between observed and expected values of stationary and mobile components were detected. When including all data (i.e., M and S intervals), 9 of the 15 tracking events from the BR study were of suitable size which led to model convergence and stable estimates of stationary and mobile components. For the stationary component, the RW model correctly predicted all nine tracking events within the 95% confidence intervals (Fig. 7a). For the mobile component, the expected values estimated from the RW model significantly overpredicted all tracking events except for tracking event 2, the first acceptable datapoint for analysis (Fig. 7b). For the TR dataset, six of the nine tracking events were retained for analysis. Stationary and mobile components of each tracking event yielded similar results to the BR study where the RW model correctly predicted each stationary component (Fig. 7c), but for the TR mobile component all tracking events were significantly overpredicted (Fig. 7d).

## Discussion

Our study provides critical information for understanding correlates between environmental, spatial, and temporal variation and the movement patterns of a megafish in need of conservation from two independent river basins. We found that longer time intervals between manual relocations of Alligator Gar dampen associations between displacement and environmental variables, and that the relationship between change of discharge and displacement is dependent on water temperature, supporting our first two hypotheses (H1-H2). Despite several studies highlighting the importance of time interval on study results [47, 48], few studies provide a quantitative assessment of these patterns. A study by [49] investigated the effect of relocation interval on Spotted Bass (*Micropterus punctulatus*). However, tracking was conducted at a diel (24-hour) scale with emphasis on habitat use rather than other environmental variables (i.e., temperature and flow) which influence movement. Leptokurtosis was observed across both BR and TR studies when data were pooled and separated by tracking event, while pooled data exhibited differences in skewness (i.e., upstream or downstream bias) between datasets. This finding supports hypotheses (H3) of the now largely accepted paradigm of leptokurtosis present in stream fishes and provides further evidence for the placement of impoundments influencing fish movement. Kurtosis was also negatively correlated with discharge conditions, but not temperature, and thus we provide partial support for H4. Lastly, the RW model developed by [17] accurately predicted the stationary component of Alligator Gar dispersal, but significantly overpredicted the mobile component. These findings contrast those of [23] and [24] where the RW model underpredicted stationary and mobile components of Leuciscid fishes. However, the life history of Alligator Gar suggests that diffusive spread does not occur due to the cyclical movement patterns of the species from spawning migrations. We therefore provide partial support for hypothesis (H5). Our findings provide further support that the movement of freshwater fishes is complex and requires an investigation of environmental, spatial, and temporal factors to fully understand movement patterns that are not accounted for in the current RW model.

For relocation observations at monthly intervals (i.e., 1–60 days), there were emergent patterns on how temperature and the change in discharge influence Alligator Gar displacement. These patterns were captured by a three-way interaction, demonstrating that movement distance was highest when the change in discharge from the previous location was largest across both studies, and when it was held at the highest temperature. These findings are similar to [50], where multispecies models of tagged fish in the Rhône River were influenced by current and antecedent hydraulic conditions, along with water temperature. However, we found that capturing these patterns were contingent on the time interval between relocations investigated, as intervals that assessed a more seasonal scale (e.g., 61–120 days) had little to no explanatory power for predicting displacement. This finding supports previous work by [25] and [34] that suggest Alligator Gar are more mobile when water temperature and discharge increases, especially during the spawning season (May-June) when water temperature is typically 20–30°C, ideal for egg incubation and larval fish development [26].

Dispersal values were also sensitive to current and antecedent discharge conditions. A clear pattern emerged while investigating average monthly discharge conditions in relation to kurtosis. Higher discharge values led to smaller kurtosis values across both studies, leading to a shift in dispersal distribution being more mesokurtic rather than leptokurtic. Considering [17] used stream size as a proxy for discharge and concluded it is positively correlated with dispersal, it is unsurprising that tracking events where average flow conditions were higher led to a higher frequency of Alligator Gar dispersal values moving away from the tagging location. Higher flows may also lead to connectivity to novel habitats that may require long distance migrations, as suggested by [35] who observed a significant proportion of Alligator Gar moving long distances to habitat that is otherwise unoccupied or unavailable. Individuals shifted from occupying deep pool mesohabitat during cooler temperatures in the Brazos River into shallow tributaries and floodplains when temperature increased, especially when temperatures coincided with higher flow conditions conducive to spawning. Similarly [34], observed seasonal shifts in mesohabitat occupied by Alligator gar in the Arkansas River basin where individuals were almost exclusively observed in the mainstem Fourche LaFave River outside of spawning season and shifted to occupying tributaries during the spawning season coinciding with flows entering flood stage. In both the BR and TR studies, mesokurtic tracking events exclusively followed flood pulses during the spawning season (May-June) with magnitudes ranging 424–1456 cubic meters per second (cms) on the Brazos River and 906 cm on the Trinity River. As corroborated by these studies, Alligator Gar displace themselves from mainstem habitats they are occupying to off-channel tributaries and floodplains, which would explain a higher frequency of movements away from the tagging location then expected under a leptokurtic distribution.

Dispersal data for both studies also lead to departures from theoretical predictions of stream fish movement. The model proposed by [17] posits a symmetrical distribution of movements, and that diffusive spread is present in riverine fish populations. However, our findings suggest that movement of Alligator Gar may not always be symmetrical and is contingent on the hydrologic scenario of the study area. Lake Livingston Dam served as the upstream boundary of the TR study, which does not possess any fish passage structures. Although downstream bias is often explained biologically, as in smolt migration of salmonids [51], we argue that anthropogenic processes are affecting Alligator Gar migration. The largest cohort of Alligator Gar tagged from the TR study was 27 individuals during the winter of 2009, of which nearly all (*n* = 23) were tagged within 30 rkm of Lake Livingston Dam. Therefore, when high flow pulses occurred, it would be more likely for their movements to be downstream rather than upstream given the sheer difference in available habitat downstream of the tagging locations. Alternatively, in the BR study individual movement was not impeded by dams on the mainstem, leading to upstream and downstream migrations for reproduction. These cyclical movements in migration would also indicate a return to respective tagging locations and therefore lead to a departure from diffusive spread. Indeed, when comparing our observed results of the mobile component of Alligator Gar dispersal to the expected results from the model developed in [17], the expected results were almost unequivocally higher than observed parameters. Furthermore, although the stationary component for the last tracking event modeled in both studies was within 95% confidence intervals, the BR stationary component was much higher than the TR stationary component. This may be explained by seasonal differences between modeled tracking events across the studies. The last modeled tracking event was in May-June for the BR study corresponding to the spawning season when Alligator Gar seasonal migrations occur and populations experience increased mobility [25, 34]. Alternatively, the final tracking event modeled from the TR study was in July during the post spawning season. Seasonal migrations are well documented in other fish taxa as well including leuciscids and percids [52, 53]. These taxa, along with Alligator Gar, are sensitive to abiotic conditions necessary for migration to occur, and therefore it would be expected that variation in temperature and flow would be more influential in affecting movement rather than time at large and diffusive spread as posited by the RW model.

Although this study provides insights into the spatial, temporal, and environmental attributes that influence stream fish movement, there are still several limitations that should be addressed. First, it should be noted that the network of streams investigated by [25] and [33] along with their study design were quite different. Nearly half of the study area in the BR study included tributaries off the Brazos River while in [25] fish were almost exclusively relocated in the mainstem Trinity River. Further, fish in the BR study were tracked primarily at monthly intervals while the TR study mainly tracked fish at seasonal (2–3 month) intervals. This led to a disparity in observations available for analysis between studies, especially given our focus on relocation intervals within a 1–60 day period. Despite this, we observed that Alligator Gar respond to temperature and flow generally in a similar manner and therefore consider these potentially confounding effects minimal. Additionally, although there were clear environmental variables driving correlations between Alligator Gar displacement and dispersal, model fit was generally low. Focusing on a shorter time interval (1–60 days) did increase the *R*^*2*^ values of our models investigating displacement, but there was still much unexplained variance. We therefore conclude that a monthly tracking interval is suitable for analysis of environmental associations to Alligator Gar movement, but finer tracking intervals may lead to stronger models than what we present here [see 35]. Future work may consider complementing finer scale manual telemetry with fixed stationary receivers, as done in marine fishes [54], to improve finer scale movement-environmental modeling of stream fish movement. Further work investigating how multiple species may respond similarly or differently to temperature and flow across a gradient of tracking intervals may lead to a more refined paradigm of the importance of environmental variables and tracking interval on predicting stream fish movement.

## Conclusions

Overall, our study demonstrates that a combination of temporal, spatial, and environmental variables are necessary to cohesively explain the dispersal and displacement of Alligator Gar. Across datasets from the two original studies, Alligator Gar appear to be responding similarly to discharge and temperature, becoming more mobile and more likely to move farther from their tagging location with increases in temperature and flow. However, these movements may exhibit bias depending on the spatial arrangement of impoundments in the study (i.e., TR study). Further, these results are only clear when the tracking interval between relocations is accounted for, where monthly intervals are adequate, and seasonal intervals are inadequate. A combination of these variables and processes in concert with the life history of Alligator Gar suggests that the RW model is not suitable for predicting Alligator Gar movement, as migratory fishes are more sensitive to environmental and spatial variation rather than time at large. In a world where freshwater megafish are disproportionately affected by human impact, it is necessary to provide critical information on the movement of individuals across riverscapes. Through our study of two independent populations of Alligator Gar, we provide a framework for other studies to follow that incorporates a complexity of processes affecting movement and provides critical information for the management of Alligator Gar by suggesting that multiple populations respond similarly to environmental change which could lead to a more general management strategy of the species across river basins.

## Electronic supplementary material

Below is the link to the electronic supplementary material.

Supplementary Material 1

Supplementary Material 2

Supplementary Material 3

Supplementary Material 4

## Data Availability

All data generated or analyzed during this study are included in this published article as supplementary files. Files include a supplementary information document, an R script, and all input data used to develop the results throughout.

## Declarations

### Disclaimer

Any use of trade, firm, or product names is for descriptive purposes only and does not imply endorsement by the U.S. Government.

## Abbreviations

RMP: Restricted movement paradigm
RW: Radinger and wolter
TL: Total length
AR: Caudal fin aspect ratio
SO: Stream order
rkm: River kilometers
°C: Degrees celsius
H1-H5: Hypotheses H1-H5
TX: Texas
BR: Brazos river
TR: Trinity river
mm: Millimeter
CART: Combined acoustic and radio-transmitter
km/h: Kilometers per hour
GPS: Global positioning system
M: Monthly interval
S: Seasonal interval
*Q*: Daily average discharge
USGS: United states geological survey
*ΔQ*: Change in daily average discharge
*z*: Z-score
h: Hour
*T*: Average daily water temperature
*ΔT*: Change in average daily water temperature
GAMs: Generalized additive models
GAM: Generalized additive model
GLMs: Generalized linear models
GLM: Generalized linear model
AICc: Akaike information criterion for small sample size
*K*: Kurtosis
*Q*_*z−30d*_: Thirty day average daily discharge.
*T*_°C−30d_: Thirty day average daily temperature.
*Z*: Z-test statistic
*p*: *P*-value
SD: Standard deviation
K: Number of parameters
cms: Cubic meters per second
