# Supplementary material for: Meta-analysis of a megafish: assessing patterns and predictors of Alligator Gar movement across multiple populations
Source: Mov Ecol. 2025 Mar 10;13:15. doi: 10.1186/s40462-025-00544-7 (PMC11892227; doi:10.1186/s40462-025-00544-7)
Supplement: Supplementary file 3 — Supplementary Material 3 [file 40462_2025_544_MOESM3_ESM.docx]

# *Any use of trade, firm, or product names is for descriptive purposes only and does not imply endorsement by the U.S. Government*

**Title: Meta-analysis of a megafish: assessing patterns and predictors of Alligator Gar movement across multiple populations**

Hayden C. Roberts^1*^, Florian J. Kappen^1^, Matthew R. Acre^3,1^, Daniel J. Daugherty^2^, Nathan G. Smith^2^, and Joshuah S. Perkin^1^

*^1^Department of Ecology and Conservation Biology, Texas A&M University, 2258 TAMU, College Station, TX 77843.*

*^2^Texas Parks and Wildlife Department, Heart of the Hills Fisheries Science Center,* *5103 Junction Highway, Mountain Home, TX 78058*

*^3^Current Address: U.S. Geological Survey, Columbia Environmental Research Center, 4200 E. New Haven Road, Columbia, MO 65201*

*^*^Correspondence:* hcr@tamu,edu*; 1 + 240 818 4964*

# **Supplementary information:**

Table S1. Summary of gages used for meta-analysis of Alligator Gar (*Atractosteus spatula*) movement. For each, the U.S. Geological Survey (USGS) gage identification number (Gage_ID) is given (U.S. Geological Survey, 2022). Additionally, the hypotheses tested using each gage, the basin each gage is from, the waterbody, and nearest town to the gages are located near are also given along with GPS coordinates. For the Navasota River Gage, OSR is abbreviated for the Old Spanish Road crossing east of Bryan, Texas.

| Gage_ID | Hypotheses | Basin | Waterbody | Location | Latitude | Longitude |
| --- | --- | --- | --- | --- | --- | --- |
| 08106500 | H1-H2 | BR | Little River | Cameron | 30.83500 | -96.94639 |
| 08110000 | H1-H2 | BR | Yegua Creek | Somerville | 30.32167 | -96.50722 |
| 08108700 | H1-H2; H4 | BR | Brazos River | Bryan | 30.62667 | -96.54389 |
| 08110800 | H1-H2 | BR | Navasota River | OSR | 30.97361 | -96.24139 |
| 08111500 | H1-H2 | BR | Brazos River | Hempstead | 30.12889 | -96.18750 |
| 08066250 | H1-H2 | TR | Trinity River | Goodrich | 30.57194 | -94.94861 |
| 08067000 | H1-H2 | TR | Trinity River | Liberty | 30.05750 | -94.81806 |
| 08066500 | H1-H2; H4 | TR | Trinity River | Romayor | 30.42500 | -94.85056 |
|  |  |  |  |  |  |  |


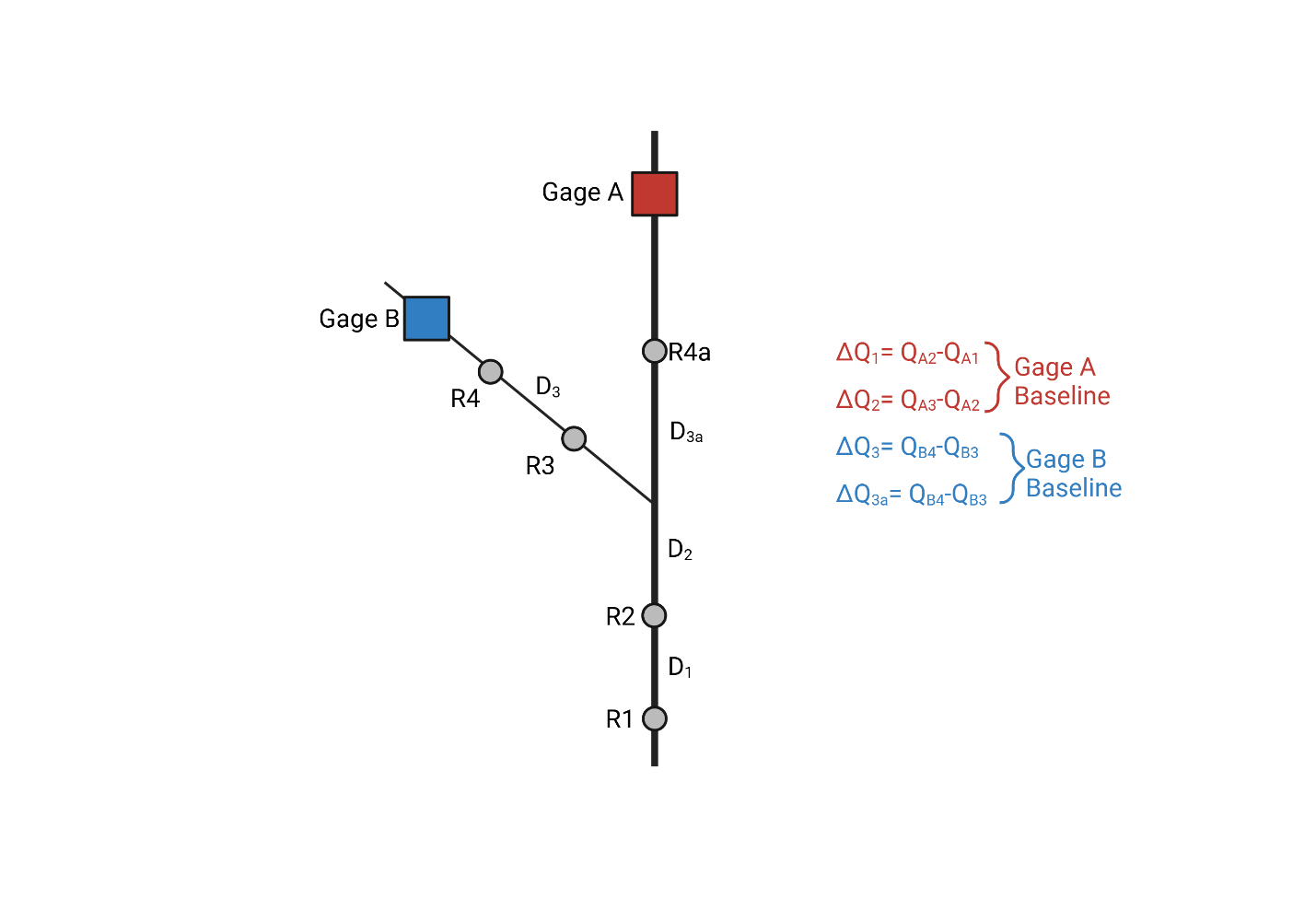


Figure S1. Conceptual diagram with hypothetical situations used to illustrate how representative gages were determined to develop relationships between Alligator Gar (*Atractosteus spatula*) displacement and the change in daily discharge (ΔQ). Relocations are abbreviated with an “R” while displacement is abbreviated “D”. For the first two relocations (R1-R2), the individual made an upstream movement on the larger mainstem river and the hydrographic distance between the relocations would be displacement distance (D_1_). The movement still left the individual downstream of Gage A (red square) and thus ΔQ would be calculated with the Gage A baseline. Between the second and third relocations (R2-R3), the individual moved into a tributary and this displacement observation (D_2_) would be the sum of the remaining hydrographic distance on the mainstem and the hydrographic distance to R3 in the tributary. In this scenario, the gage baseline would still be Gage A under the assumption that ΔQ on the mainstem (e.g., rise in flow magnitude) led to a movement into the tributary. For the last two relocations (R3-R4), the fish remained in the tributary and thus Gage B (blue square) would be used as the discharge baseline and D_3_ would be the hydrographic distance between R3 and R4. Alternatively, if the individual moved from the tributary back to the mainstem (R3-R4a), Gage B would still be the baseline as ΔQ (e.g., fall in flow magnitude) may elicit a movement out of the tributary back to the mainstem. The displacement observation for this scenario (D_3a_) would be calculated the same as (D_2_). Figure created with BioRender.com (<https://biorender.com/>).


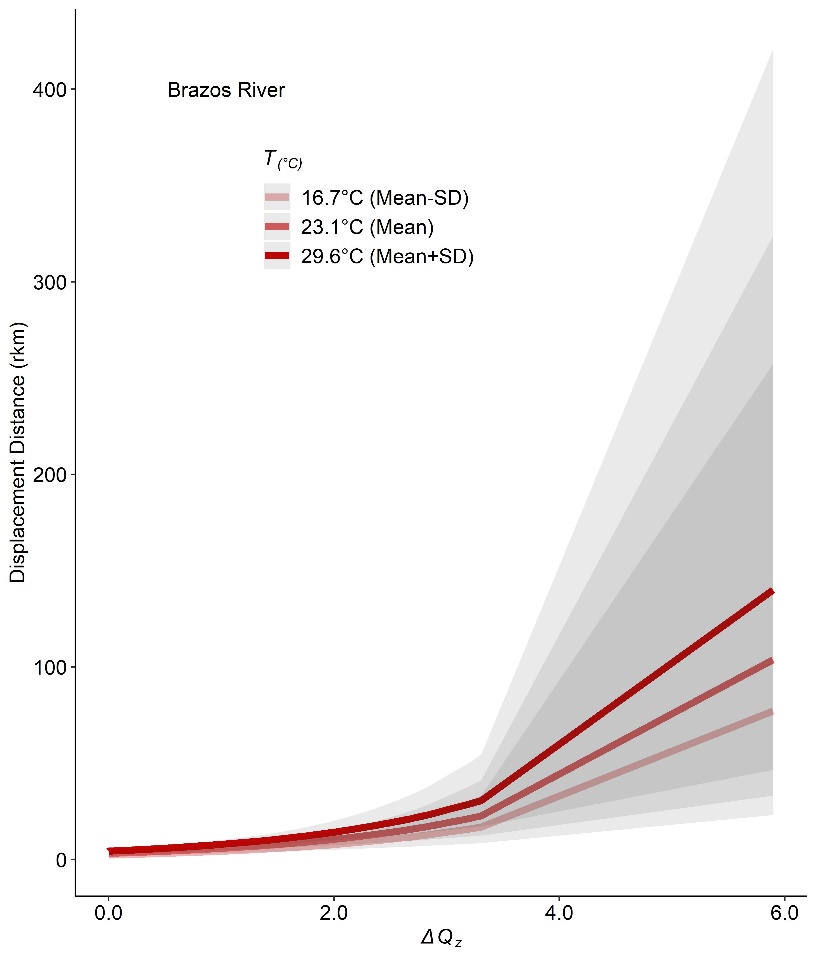


Figure S2. Partial effects plot from top model of our hypotheses investigating environmental predictors of Alligator Gar (*Atractosteus spatula*) displacement when all observations of Fish 138 are included. When including these observations, the top model also included a three-way interaction between the change in z-score transformed daily average discharge *ΔQ_z_*, daily average temperature, and the *study* identity of each observation. Note when *ΔQ_z_* approaches 4, the 95% confidence intervals become increasingly uncertain as the only observations exceeding this value stem from the two observations of Fish 138 exceeding 130 rkm. See Figure 4 in the main text for a comparison of confidence interval estimates.


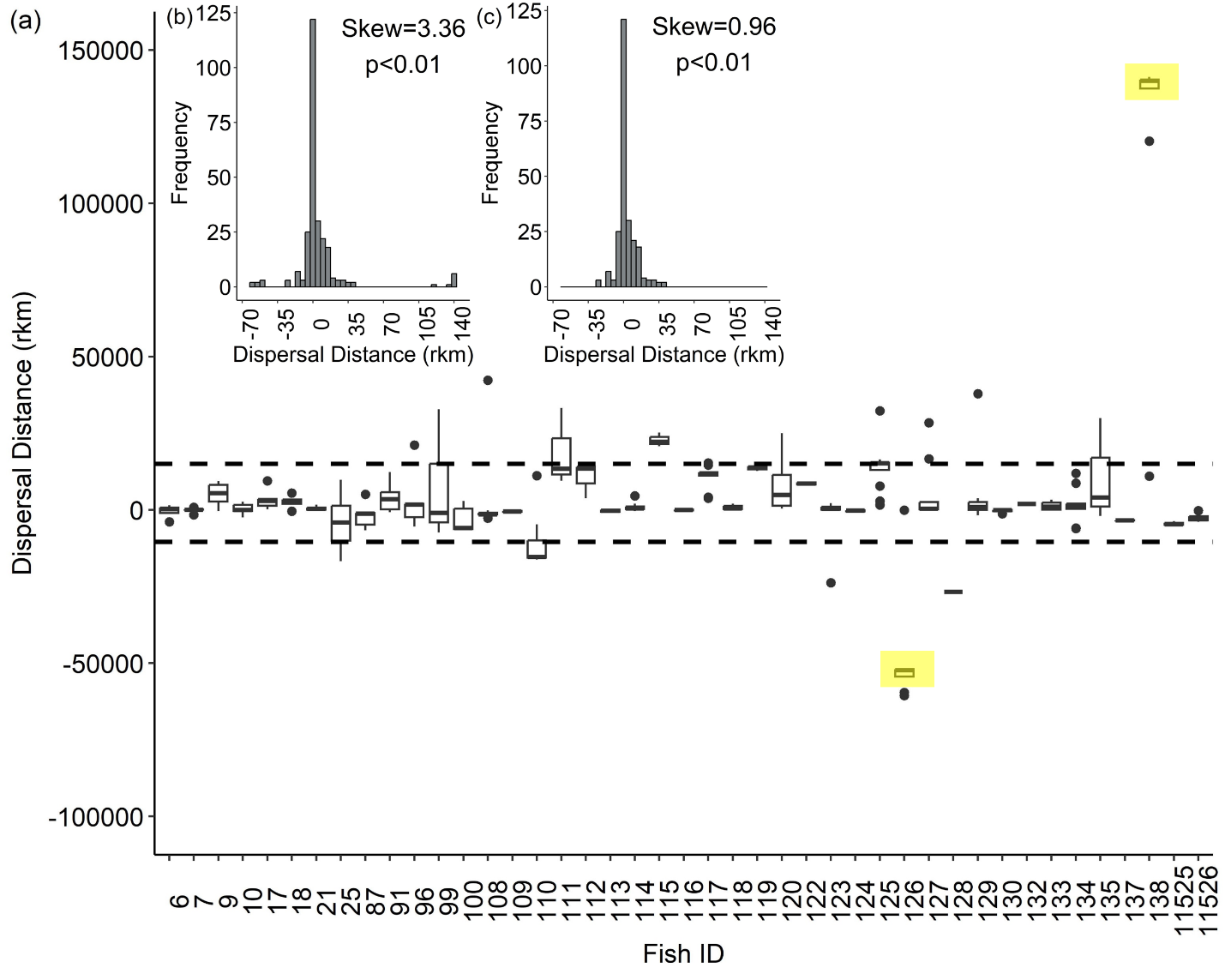


Figure S3. Summary of the distribution of movements for all Alligator Gar (*Atractosteus spatula*) with at least one relocation tagged in the Brazos River study (Roberts et al. 2022), illustrated with boxplots and histograms. In (a), the upper and lower dashed lines represent the threshold for observations considered outliers. Each box blot represents the distribution of dispersal movements of a tagged Alligator Gar with a unique tag identifier (Fish ID). In (b), a histogram demonstrates the positive skewness (skew) of dispersal movements of all Alligator Gar. In (c), the same histogram is illustrated, however, Fish 126 and Fish 138 were removed as outliers, and are highlighted in yellow boxes in (a).


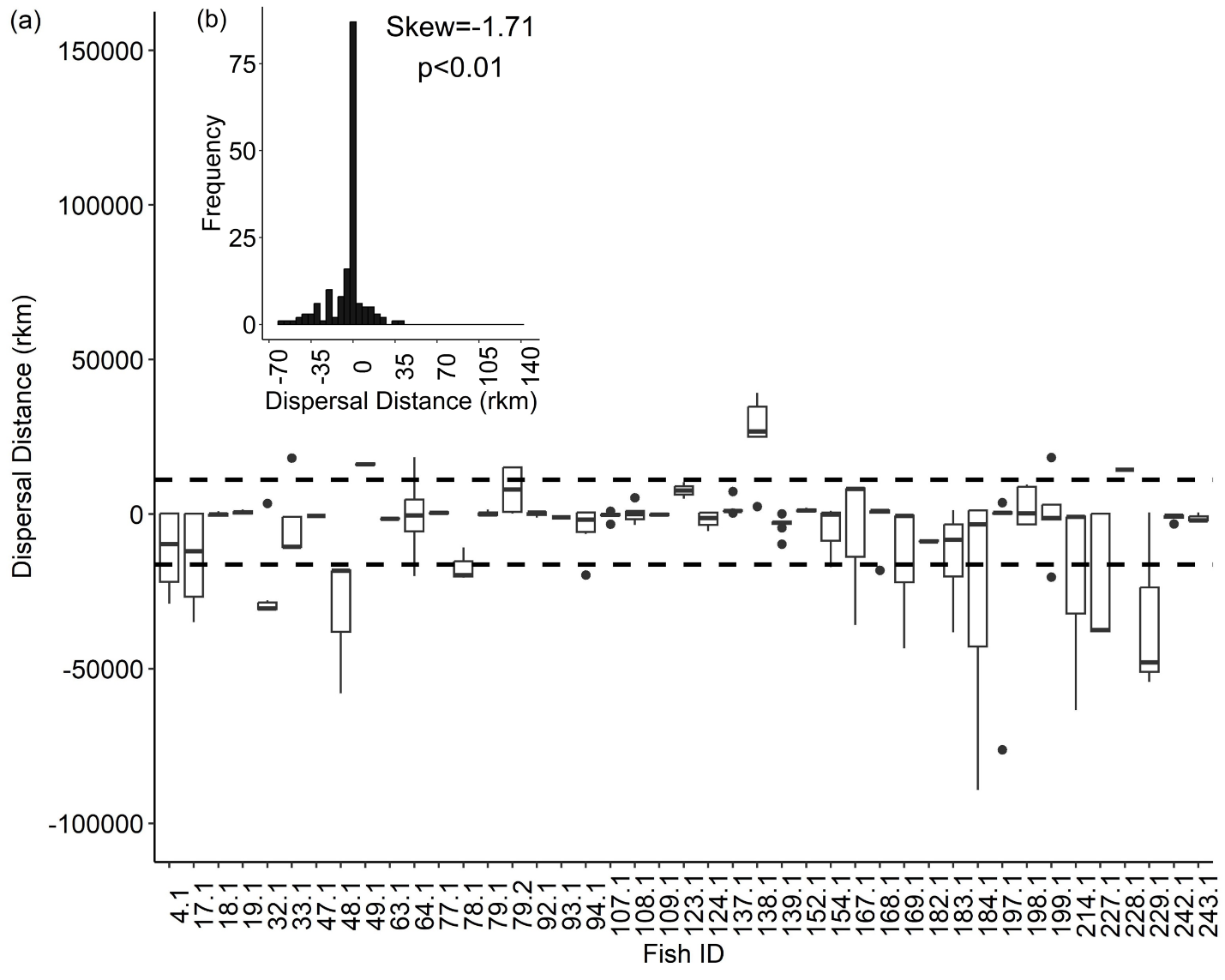


Figure S4. Summary of the distribution of movements for all Alligator Gar (*Atractosteus spatula*) tagged in the Trinity River study (Buckmeier et al. 2013) with at least one relocation illustrated with boxplots and histograms. In (a), the upper and lower dashed lines represent the threshold for observations considered outliers. Each box blot represents the distribution of dispersal movements of a tagged Alligator Gar with a unique tag identifier (Fish ID). In (b), a histogram demonstrates the negative skewness (skew) of dispersal movements of all Alligator Gar.


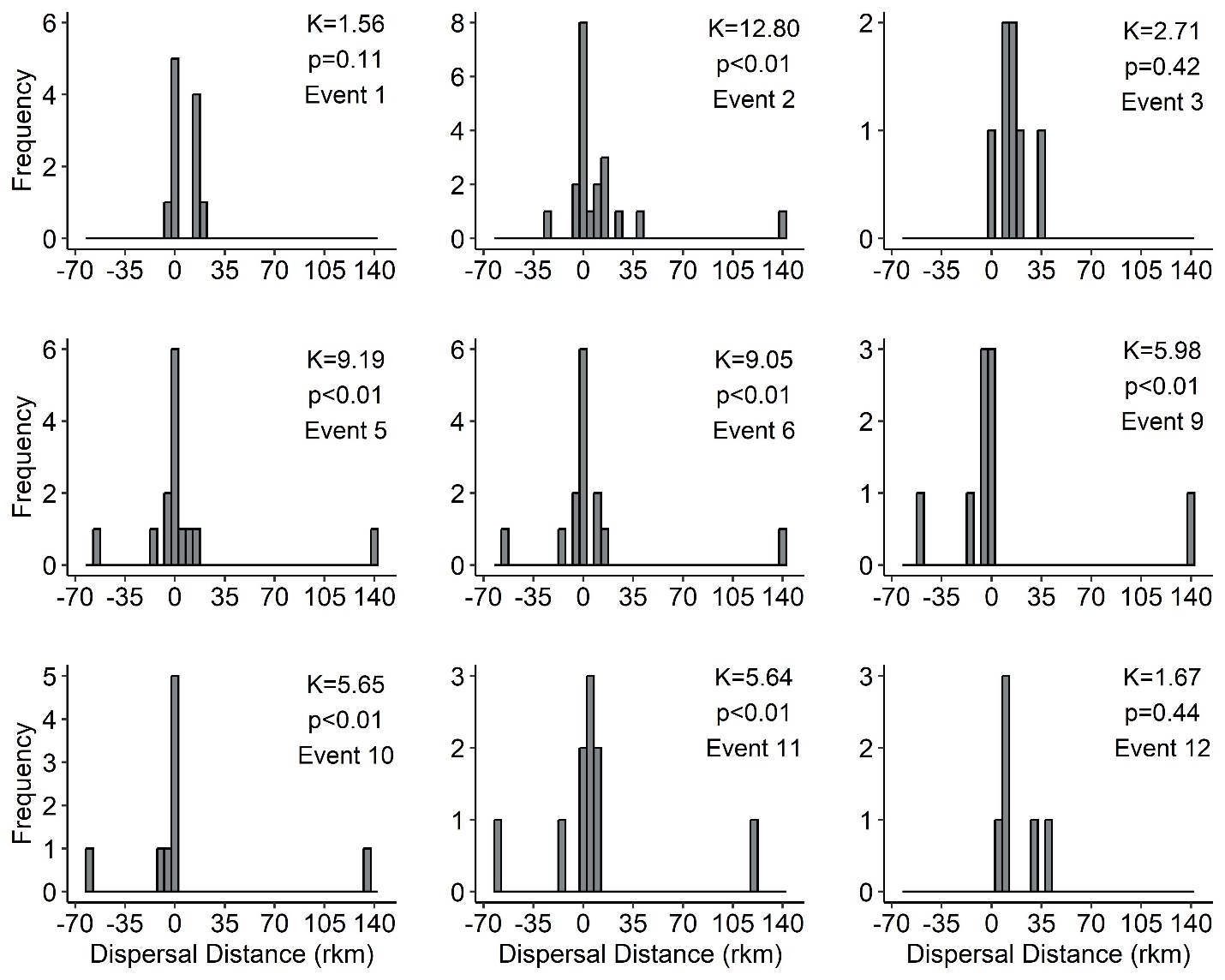


Figure S5. Frequency distributions for each tracking event to assess Alligator Gar (*Atractosteus spatula*) dispersal and its relationship with environmental variables for the Brazos River. Data are from a subset of tagged individuals (n=23) with observations from the monthly time interval (i.e., 1-60 day relocation interval). The kurtosis value (K) for each tracking event is given in the upper right-hand corner, along with associated p-values. A K value significantly higher than three suggests the frequency distribution is leptokurtic.


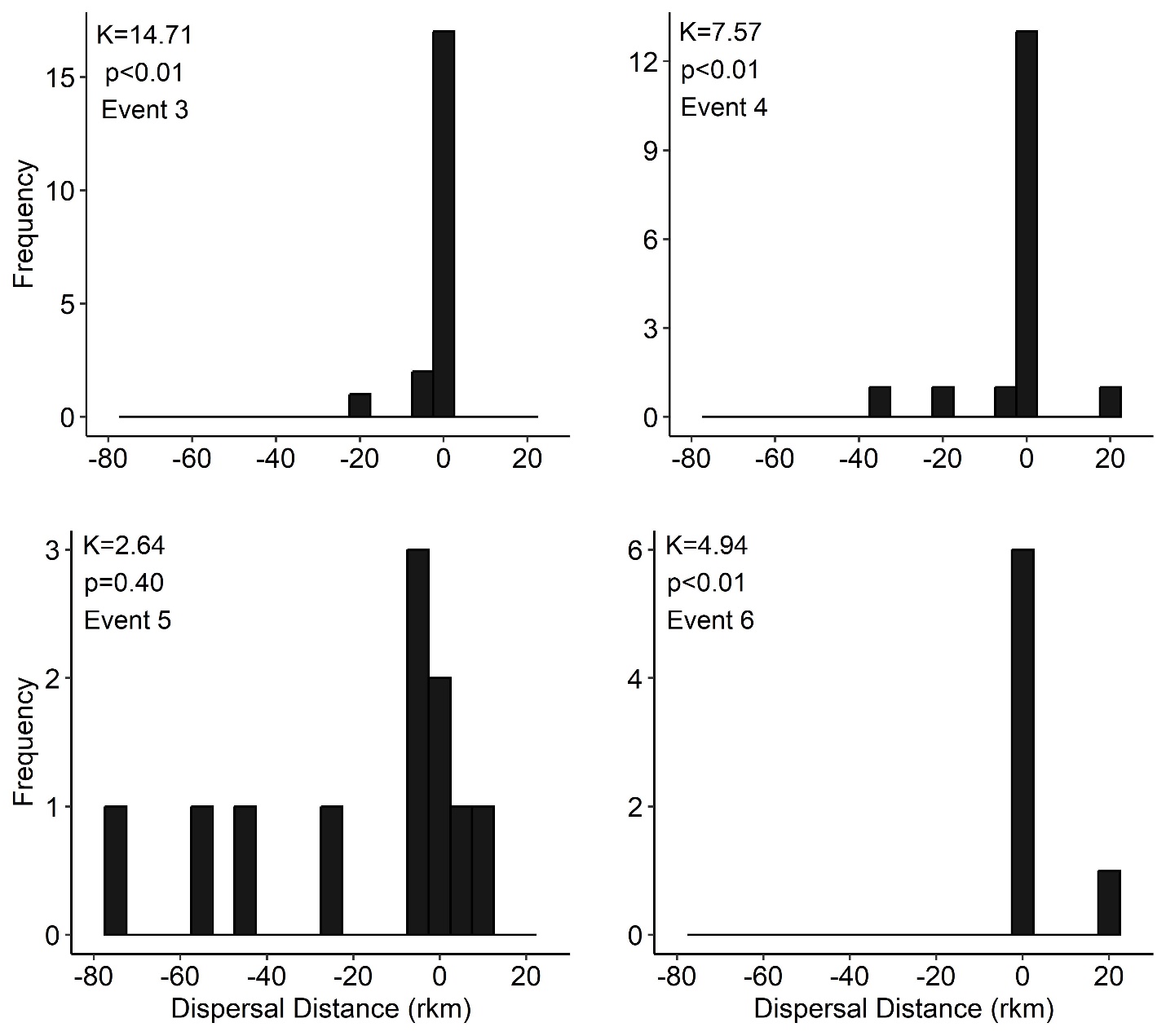


Figure S6. Frequency distributions for each tracking event to assess Alligator Gar (*Atractosteus spatula*) dispersal and its relationship with environmental variables for the Trinity River. Data are from a subset of tagged individuals (n=27) with filtered observations from the monthly interval (i.e., 1-60 day relocation interval). The kurtosis value (K) for each tracking event is given in the upper left-hand corner, along with associated p-values. A K value significantly more than three suggests that the frequency distribution is leptokurtic.

References

- Buckmeier, D. L., Smith, N. G., & Daugherty, D. J. (2013). Alligator Gar movement and macrohabitat use in the lower Trinity River, Texas. *Transactions of the American Fisheries Society*, *142*(4), 1025-1035.
- Roberts, H. C., Acre, M. R., Claus, M. P., Kappen, F. J., Winemiller, K. O., Daugherty, D. J., & Perkin, J. S. (2022). Tributary streams provide migratory fish with access to floodplain habitats in a regulated river: evidence from alligator gar, Atractosteus spatula. *Canadian Journal of Fisheries and Aquatic Sciences*, *80*(2), 393-407.
- U.S. Geological Survey, 2022, USGS water data for the Nation: U.S. Geological Survey National Water Information System database, accessed August 20, 2022, at https://doi.org/10.5066/F7P55KJN.
